# Risk factors and outcomes of post-traumatic endophthalmitis: a retrospective single-center study

**DOI:** 10.1186/s12348-021-00254-2

**Published:** 2021-08-02

**Authors:** Nawat Watanachai, Janejit Choovuthayakorn, Susama Chokesuwattanaskul, Chaipot Photcharapongsakul, Praelada Wongsirimeteekul, Phichayut Phinyo, Voraporn Chaikitmongkol, Paradee Kunavisarut, Pongsant Supreeyathitikul, Direk Patikulsila

**Affiliations:** 1 Department of Ophthalmology, Faculty of Medicine, Chiang Mai University, 110 Intavaroros Road, Maung, Chiang Mai, 50200 Thailand; 2 Clinical Epidemiology and Clinical Statistics Center, Faculty of Medicine, Chiang Mai University, Chiang Mai, Thailand; 3 Department of Family Medicine, Faculty of Medicine, Chiang Mai University, Chiang Mai, Thailand; 4 Musculoskeletal Science and Translational Research (MSTR), Chiang Mai University, Chiang Mai, Thailand

## Abstract

**Background:**

To describe the epidemiology, characteristics, risk factors, and outcomes of post-traumatic endophthalmitis.

**Main body:**

Medical records of consecutive open globe injury patients admitted and primarily treated between January 2006 and December 2016 were retrospectively reviewed. Patients were defined as having or not having associated endophthalmitis. Data of demographics, injury characteristics, clinical presentations, and visual outcomes were collected. The potential risks and significant factors for visual outcomes of post-traumatic endophthalmitis were determined. There were 591 patients included in this study. Among these, 118 patients were clinically diagnosed as having accompanied endophthalmitis. Higher proportions of intraocular foreign body (IOFB) (55.1% vs. 27.3%) and injury related to high-velocity objects (55.9% vs. 32.6%) were noted in patients with endophthalmitis compared to patients without endophthalmitis. Anterior wound location (odds ratio [OR], 2.0; 95% confidence interval [CI], 1.1 to 3.7; *P* = 0.020), presence of IOFB (OR, 1.9; 95% CI 1.2 to 3.0; *P* = 0.005), and delayed presentation of > 24 h (OR, 3.9; 95% CI 2.3 to 6.4; *P* < 0.001) were significant risk factors for associated endophthalmitis. Final visual acuity (VA) of the overall population improved significantly from 2.4 (0.6) logMAR to 1.4 (0.1) logMAR, *P* < 0.001, however, patients in the endophthalmitis group achieved a worse final VA than the non-endophthalmitis group (66.1% vs. 43.5%, *P* < 0.001).

**Conclusion:**

High proportions of post-traumatic endophthalmitis patients had subsequent poor visual outcomes. Therefore, safety and protective measurements, especially when performing activities related to high-velocity objects, and the institution of prophylactic antibiotics in high-risk groups should be promptly considered to reduce the incidence.

## Introduction

Ocular trauma, particularly open globe injury (OGI), is one of the major causes of acquired visual loss of the general population at all ages. Visual disability after ocular trauma can be determined by several factors, including mechanism of injury, the severity of damaged ocular tissue, and associated complications [1–7]. One of the most deleterious complications is post-traumatic endophthalmitis, which is caused by inoculation of pathogens, either normal ocular flora or other environmental microorganisms, through a breakdown of a corneoscleral shell into an eyeball. Commonly, the condition results in profound visual loss following OGI. Compared to other intraocular infections (such as post-operative endophthalmitis), post-traumatic endophthalmitis patients had worse final visual outcomes [8–10]. Therefore, a close monitoring of cases with high risk for endophthalmitis, early recognition, and prompt management are crucial steps to decrease the incidence and improve the visual outcomes among these patients.

This study aimed to describe the etiology, clinical characteristics, and outcomes of patients with post-traumatic endophthalmitis treated at a tertiary center in Northern Thailand. Information may provide additional insights for clinicians regarding prevention, management, and visual prognosis of the condition.

## Methods

This observational study was conducted in accordance with the tenets of the Declaration of Helsinki. The protocol was approved by the University Research Ethics Committee. Due to anonymized data, informed consent was exempted. Patients with OGI, defined as a full-thickness laceration of either cornea or sclera according to The Birmingham Eye Trauma Terminology System (BETT), admitted at Chiang Mai University hospital between January 2006 and December 2016 were identified [11]. Medical records of consecutive patients who admitted for primary ophthalmic management at this hospital and followed for at least 1 month were reviewed. In a protocol-specific record form, demographics including age, gender, activity and mechanism of injury, and time interval from injury to ophthalmic evaluation were collected. Besides, details of globe injury including laterality, initial visual acuity (VA), anterior and posterior segment abnormalities, and imaging investigations (B-scan ultrasonography, skull film, or computerized tomography) were assessed. Wound location was defined as zone I (laceration limited to the cornea), zone II (laceration involving the sclera, within 5-mm of the corneoscleral limbus), and zone III (laceration involving posterior sclera, beyond 5-mm of the corneoscleral limbus) [12]. Presence of endophthalmitis was determined by treating retinal physicians (DP, JC, NW, PK, and VC) using the following clinical symptoms and signs: eye pain and redness; deteriorating in VA; purulent discharge; chemosis; eyelid edema; fibrin and/or hypopyon in the anterior chamber; and vitreous opacification.

For medical management, all OGI patients immediately received empirical intravenous vancomycin 1 g every 12 h and ceftazidime 1 g every 8 h (starting at presentation to this hospital or before if referred from the outside centers) and continued for 3 to 5 days. For patients who presented and/or developed symptoms and signs of endophthalmitis as well as patients who presented with high risk of infection (delayed presentation and having intraocular foreign body, IOFB), intravitreal injections of vancomycin 1 mg in 0.1 ml and ceftazidime 2.25 mg in 0.1 ml were administered. Additionally, in patients with evidence of fungus infection from the intraocular fluid investigation, an intravitreal amphotericin B (5 μg in 0.1 ml) was injected. The application of intensive topical antibiotics (cefazolin 33 mg/ml and gentamicin 14 mg/ml), 1% prednisolone acetate, and an oral systemic antibiotic (ciprofloxacin 500 mg twice a day) were considered by treating physicians based on the severity of the infection and associated ocular injuries. Any surgical management, including pars plana vitrectomy (PPV), was reviewed. In endophthalmitis patients, microbiological results from both aqueous and vitreous fluid samplings (which were examined and cultured on conventional media including blood agar, chocolate agar, thioglycolate broth, and Sabouraud dextrose agar) were collected. Final anatomical and visual outcomes at the last follow-up visit were recorded.

### Statistical analysis

Categorical data were described as frequency and percentage, while continuous data as mean (standard deviation, SD) or median (interquartile range, IQR). Snellen VA was calculated into a logarithm of the minimum angle of resolution (logMAR) unit for the statistical analysis. Patients were categorized into OGI with accompanied intraocular infection (endophthalmitis group) and OGI without the intraocular infection (non-endophthalmitis group). In comparative statistical analysis, categorical data were assessed by Fisher’s exact or Chi-squared test, while independent T-test or non-parametric test were used for continuous variables. Risk of endophthalmitis (dependent variable) was estimated by multivariable logistic regression. Independent variables included in a model were factors that showed significant differences in univariable calculation. In addition, prognostic factors associated with poor visual outcome (defined as final VA of less than 20/400) among endophthalmitis patients were also evaluated in a model that included age, gender, initial VA, wound characteristics (location, self-sealing, and contamination), presence of relative afferent pupillary defect (RAPD), retinal detachment, presence of IOFB, vitreous and uveal tissue prolapse, positive culture results, performing PPV, and time from injury to hospital as independent factors. Data were analyzed by the SPSS version 16.0 software (SPSS Inc, Chicago, IL, USA). A *P* value of less than 0.05 was considered a statistical significance.

## Results

A total of 591 OGI patients (591 eyes) with a median (IQR) follow-up of 5.5 (2 to 17) months were included in this study. Three hundred and seventy-two patients (62.9%) were referred from outside centers. Of all patients, 118 were clinically diagnosed with endophthalmitis. The demographics and injury characteristics of overall OGI patients categorized by accompanying endophthalmitis are summarized in Table 1. Overall, there were no differences in mean age (*P* = 0.655), gender (*P* = 0.747), and laterality (*P* = 0.303) between patients with and without endophthalmitis. However, patients in the endophthalmitis group tended to have sustained injuries related to IOFB (55.1% vs. 27.3%) and high-velocity objects (55.9% vs. 32.6%), and lived in rural areas (76.3% vs. 56.9%) compared to patients in the non-endophthalmitis group.

**Table 1:** Demographics and injury characteristics of open globe injury patients divided into endophthalmitis and non-endophthalmitis groups

Characteristics	Total (*N* = 591)	Endophthalmitis group (*N* = 118)	Non-endophthalmitis group (*N* = 473)	*P* value
Mean age (SD), year	39.0 (18.2)	39.2 (17.2)	38.9 (18.4)	0.655
Gender (male/female)	524/67	106/12	418/55	0.747
Laterality (right/left)	298/293	54/64	244/229	0.303
Age group (year), n (%)				0.246
0 to 20	105 (17.8)	18 (15.3)	87 (18.4)	
> 20 to 40	200 (33.8)	39 (33.1)	161 (34.0)	
> 40 to 60	225 (38.1)	53 (44.9)	172 (36.4)	
> 60	61 (10.3)	8 (6.8)	53 (11.2)	
Mechanism of injury, n (%)				< 0.001
IOFB	194 (32.8)	65 (55.1)	129 (27.3)	
Penetration/perforation	287 (18.6)	49 (41.5)	238 (50.3)	
Rupture	110 (48.6)	4 (3.4)	106 (22.4)	
Injury site, n (%)				< 0.001
Workplace based	375 (63.5)	96 (81.4)	279 (59.0)	
Outdoor based	181 (30.6)	18 (15.3)	163 (34.5)	
Home and indoor based	35 (5.9)	4 (3.4)	31 (6.6)	
Object causing injury, n (%)				< 0.001
Mowing related projectile objects	147 (24.9)	53 (44.9)	94 (19.9)	
Chiseling/hammering related projectile objects	73 (12.4)	13 (11.0)	60 (12.7)	
Other metallic objects	59 (10.0)	16 (13.6)	43 (9.1)	
Sticky wood/wooden object	90 (15.2)	15 (12.7)	75 (15.9)	
Explosive object	54 (9.1)	7 (5.9)	47 (9.9)	
Hit by other blunt objects	78 (13.2)	5 (4.2)	73 (15.4)	
Needle/knife	20 (3.4)	4 (3.4)	16 (3.4)	
Glass	55 (9.3)	2 (1.7)	53 (11.2)	
Unknown	15 (2.5)	3 (2.5)	12 (2.5)	
Address of injury, n (%)				< 0.001
Rural	359 (60.7)	90 (76.3)	269 (56.9)	
Urban	232 (39.7)	28 (23.7)	204 (43.1)	
Soil/vegetation contaminated injury, n (%)	237 (40.1)	68 (57.6)	169 (37.5)	< 0.001

*IOFB* intraocular foreign body, *SD* standard deviation

### Clinical characteristics

For a mean (SD) presenting VA, patients in the endophthalmitis group had poorer vision than patients without endophthalmitis [2.3 (0.7) logMAR unit vs. 2.0 (0.9) logMAR unit, *P* < 0.001). Wound characteristics (location, contamination, and self-sealing], and vitreous and uveal tissue prolapse were significantly different between the two groups (all *P* values < 0.001). Considering the time interval from injury to the hospital, a median (IQR) of 24 (7 to 90) hours was observed for the overall cases. However, the proportions of patients with delayed presentation > 24 h and the patients who received primary surgery > 24 h were significantly higher in the endophthalmitis group, compared to the non-endophthalmitis group (78.8% vs. 40.4%, *P* < 0.001 and 94.9% vs. 65.0%, *P* < 0.001, respectively). Among 284 patients who had a delayed presentation (with subsequent delayed surgical interventions), the attributable factors included a lack of awareness to visit a healthcare professional after eye injury (209, 73.6%), difficulty in travelling and accessing the healthcare facility (45, 15.8%), a delayed diagnosis (19, 6.7%), and undetermined in 11 (3.4%) patients. Table 2 demonstrates the detailed clinical manifestations of OGI patients in the endophthalmitis and non-endophthalmitis groups.

**Table 2:** Clinical presentations of open globe injury patients by endophthalmitis groups

Clinical characteristics	Total (*N* = 591)	Endophthalmitis group (*N* = 118)	Non-endophthalmitis group (*N* = 473)	*P* value
Wound location, n (%)				< 0.001
Zone I	281 (47.5)	80 (67.8)	201 (42.5)	
Zone II	166 (28.1)	21 (17.8)	145 (30.7)	
Zone III	144 (24.4)	17 (14.4)	127 (26.8)	
Lens capsule rupture, n (%)	104 (17.6)	24 (20.3)	80 (16.9)	0.422
Vitreous prolapsed, n (%)	138 (23.4)	10 (8.5)	128 (27.1)	< 0.001
Uveal tissue prolapsed, n (%)	261 (44.2)	34 (28.8)	227 (48.0)	< 0.001
Retinal detachment, n (%)	133 (22.5)	26 (22.0)	107 (22.6)	0.904
Choroidal detachment, n (%)	72 (12.2)	8 (6.8)	64 (13.5)	0.063
RAPD, n (%)	184 (31.1)	36 (30.5)	148 (31.3)	0.912
Presenting to hospital > 24 h, n (%)	284 (48.1)	93 (78.8)	191 (40.4)	< 0.001
Median duration of admission (IQR), day	9 (6 to 12)	10 (7 to 14)	8 (6 to 12)	< 0.001
Mean final (SD) VA, logMAR^a^	1.4 (1.1)	1.9 (1.0)	1.3 (1.1)	< 0.001
Final VA worsen than 20/400, n (%)^a^	282 (48.0)	78 (66.1)	204 (43.5)	< 0.001

*RAPD* relative afferent pupillary defect, *IQR* interquartile range, *VA* visual acuity, *SD* standard deviation, *logMAR* logarithm of the minimum angle of resolution, *FVA* VA at final follow-up visit

^a^VA at final follow-up could not be determined in 4 patients of non-endophthalmitis group

### Risks factors for endophthalmitis

In multivariable analysis, factors associated with development of endophthalmitis following OGI were Zone I wound location (odds ratio [OR], 2.0; 95% confidence interval [CI], 1.1 to 3.7; *P* = 0.020), presence of IOFB (OR, 1.9; 95% CI 1.2 to 3.0; *P* = 0.005), and delayed presentation > 24 h (OR, 3.9; 95% CI 2.3 to 6.4; *P* < 0.001) (Table 3). The probabilities of endophthalmitis in patients with these factors are shown in Table 4.

**Table 3:** Multivariable regression for risk of endophthalmitis development following open globe injury by full analysis and reduced model analysis

Variables	Full analysis			Backward LR		
	Odds ratio	95% CI	*P* value	Odds ratio	95% CI	*P* value
Wound location						
Zone III	–	–	–	–	–	–
Zone II	1.3	0.6–2.6	0.521	1.1	0.6–2.3	0.701
Zone I	1.9	1.1–3.5	0.045	2.0	1.1–3.7	0.020
Vitreous prolapse	1.8	0.9–3.9	0.124	NA		
Uveal tissue prolapse	0.8	0.5–1.4	0.510	NA		
Presence of IOFB	1.6	0.9–2.6	0.078	1.9	1.2–3.0	0.005
Self-sealed wound	1.6	0.9–2.7	0.107	NA		
Contaminated wound	1.3	0.8–2.1	0.248	NA		
Rural address	0.9	0.6–1.7	0.984	NA		
Time to hospital > 24 h	3.3	1.8–5.9	< 0.001	3.9	2.3–6.4	< 0.001

*CI* confidence interval, *IOFB* intraocular foreign body

**Table 4:** Probability of endophthalmitis development following open globe injury by associated risk characteristics

	Probability of endophthalmitis (%)								
	2 factors						3 factors		
Wound location									
Zone 3	**×**			**×**			**×**		
Zone 2		**×**			**×**			**×**	
Zone 1			**×**			**×**			**×**
Presence of IOFB	**×**	**×**	**×**				**×**	**×**	**×**
Time to hospital > 24 h				**×**	**×**	**×**	**×**	**×**	**×**
	9.5	10.8	17.6	17.5	19.6	30.1	28.9	31.8	45.2

Receiver operating curve (ROC) = 0.79

*IOFB* intraocular foreign body

### Microbiological proven

For 118 endophthalmitis patients, aqueous and vitreous fluid collected at presentation and/or at beginning of the operations were subjected for microbiological analysis (but not for fluid obtained from a vitrectomy cassette). Microbiological etiologies were confirmed in 25 (21.2%) patients (2 had positive results from aqueous and vitreous cultures and 23 from vitreous cultures only). Endophthalmitis patients with positive cultures had a lesser proportion of a self-sealing wound (24% vs. 49.5%, *P* = 0.040), a higher proportion of enucleation/evisceration (40% vs. 15.1%, *P* = 0.013, and worse final VA (88% vs. 60.2%) compared to negative culture patients (Table 5). Table 6 describes the microbiological results of endophthalmitis patients with positive cultures. The most common microbiological spectrum identified was gram-positive organism (including *Bacillus* spp. and coagulase-negative *Staphylococcus*). Mixed organisms were cultured in 3 cases and fungus in 1 patient.

**Table 5:** Characteristics of endophthalmitis patients by culture results (*N* = 118)

Characteristics	Endophthalmitis (*N* = 118)		*P* value
	Positive culture (*N* = 25)	Negative culture (*N* = 93)	
Presence of IOFB, n (%)	15 (60.0)	50 (53.8)	0.741
Lens capsule rupture, n (%)	8 (32.0)	16 (17.2)	0.176
Presenting to hospital > 24 h, n (%)	17 (68.0)	76 (81.7)	0.224
Self-sealing wound, n (%)	6 (24.0)	46 (49.5)	0.040
Mean initial (SD) VA, logMAR	2.4 (0.7)	2.3 (0.7)	0.382
Receiving intravenous antibiotics prior presentation, n (%)	19 (76.0)	77 (82.8)	0.627
Final VA worsen than 20/400, n (%)^a^	22 (88.0)	56 (60.2)	0.018
Enucleation/evisceration, n (%)	10 (40.0)	14 (15.1)	0.013

*VA* visual acuity, *SD* standard deviation, *logMAR* logarithm of the minimum angle of resolution, *IOFB* intraocular foreign body

^a^VA at final follow-up could not be determined in 4 patients of non-endophthalmitis group

**Table 6:** Microbiological distribution of culture-positive post-traumatic endophthalmitis patients (*N* = 25)

Microbiological proven	Number
Gram positive (*N* = 18)	
*Bacillus species*	7
*Staphylococcus coagulase negative*	4
*Streptococcus pneumoniae*	3
*Staphylococcus epidermidis*	2
*Streptococcus viridan*	1
*Staphylococcus aureus*	1
Gram negative (*N* = 3)	
*Enterobacter cloacae*	1
*Serratia marcescens*	1
*Escherichia coli*	1
Mixed organism (*N* = 3)	
*Streptococcus pneumoniae/Bacillus* species	1
*Enterococcus faecalis/Pantoea agglomerans*	1
*Escherichia coli/ Enterobacter cloacae*	1
Fungus (*N* = 1)	
*Paecilomyces* species	1

### Treatments and outcomes

Intravenous antibiotics treatments were given prior to the presentation to our hospital in 372 referral patients (96 diagnosed as endophthalmitis and 276 non-endophthalmitis) and at the time of presentation in 219 patients who were primarily managed at this hospital. No one received the intravitreal antibiotic injections before the presentation. However, for 118 OGI patients with endophthalmitis and 90 OGI patients with high-risk characteristics for infection (delayed presentation and retained IOFB), the injections were performed at the presentation to this hospital. Of 591 OGI patients, 454 (76.8%) required primary wound repair, whereas 137 (23.2%) had a self-sealing wound. Characteristics of patients with self-sealing wounds are described in Table 7. Of note, a higher distribution of zone I injury, presence of IOFB, endophthalmitis, and delayed presentation to the hospital were observed in patients with a self-sealing wound compared to patients requiring primary wound repair (*P* values < 0.001). In this study, none of the patients without presenting endophthalmitis developed the infection during treatments and follow-up.

**Table 7:** Presentations and outcomes of open globe injury divided into patients with primary self-sealing wound and patients requiring primary wound repair

Characteristics	Open globe injury (*N* = 591)		*P* value
	Self-sealing wound (*N* = 137)	Non-self-sealing wound (*N* = 454)	
Workplace based injury, n (%)	116 (84.7)	259 (57.0)	< 0.001
Projectile-related objects (mowing and chiseling), n (%)	76 (55.5)	144 (31.8)	< 0.001
Zone I injury, n (%)	92 (67.2)	189 (41.6)	< 0.001
Presence of IOFB, n (%)	100 (73.0)	94 (20.7)	< 0.001
Presence of endophthalmitis, n (%)	52 (38.0)	66 (14.5)	< 0.001
Lens capsule rupture, n (%)	17 (12.4)	87 (19.2)	0.091
Presenting to hospital > 24 h, n (%)	114 (83.2)	170 (37.4)	< 0.001
Mean initial (SD) VA, logMAR	1.8 (0.9)	2.4 (0.6)	< 0.001
Final VA worsen than 20/400, n (%)^a^	56 (40.9)	226 (50.2)	0.088

*VA* visual acuity, *SD* standard deviation, *logMAR* logarithm of the minimum angle of resolution, *IOFB* intraocular foreign body

^a^VA at final follow-up could not be determined in 4 patients of non-endophthalmitis group

For overall OGI, the proportions of patients who underwent PPV or enucleation/evisceration were higher in the endophthalmitis compared to the non-endophthalmitis group [110 (93.2%) vs. 230 (48.6%), *P* < 0.001 and 24 (20.3%) vs. 59 (12.5%), *P* = 0.04, respectively]. Among 24 endophthalmitis patients who underwent enucleation/evisceration, 10 had positive culture results [6 out of all 7 patients with *Bacillus species* infection, 2 of all 3 patients with gram negative organism (*Serratia marcescens* and *Escherichia coli*) infection, and 2 of all 3 patients with mixed-organism infection]. A mean (SD) final VA of the overall OGI population significantly improved from 2.4 (0.6) logMAR to 1.4 (0.1) logMAR, *P* < 0.001. However, patients in the endophthalmitis group achieved a worse final VA than in the non-endophthalmitis group (*P* < 0.001). For endophthalmitis patients, presence of RAPD (OR, 9.5; 95% CI 3.1 to 61.4; *P* = 0.024), and positive microbiological culture (OR, 4.7; 95% CI 1.2 to 21.5; *P* = 0.044) were indicated factors for poor final visual outcome in the multivariable analysis. Notably, all three endophthalmitis patients with mixed organisms had a final VA worse than 20/400. During the follow-up, 14 patients in the endophthalmitis group developed hypotony compared to 29 patients in the non-endophthalmitis group (11.9% vs. 6.1%, *P* = 0.014), 13 vs. 41 patients for retinal detachment (11.0% vs. 8.7%, *P* = 0.181), and 8 vs. 34 patients for secondary glaucoma (6.8% vs. 7.2%, *P* = 0.892).

## Discussion

In this study, a risk of post-traumatic endophthalmitis among OGI patients is associated with anterior wound location, presence of IOFB, and delay in primary wound closure. Even though vision significantly improved after OGI treatments, an unfavorable visual prognosis was more frequently observed in the endophthalmitis group than in the non-endophthalmitis group.

Infectious endophthalmitis is a severe ophthalmic condition developed from several etiologies such as ocular trauma, previous intraocular surgery, hematologic spreading, and associated corneal ulceration. Therefore, different etiologies contained different incidences, characteristics, and visual outcomes [13–15]. For post-traumatic endophthalmitis, occurrence and visual prognosis are associated with the nature and extent of the injury, the timing of diagnosis and management, and virulence of inoculated microorganisms [16–21]. Among these, the recognition of high-risk injury settings is necessary to effectively facilitate a specific prevention program. From previous reports, the situations related to post-traumatic endophthalmitis were varied (e.g. mostly associated with trauma caused by needles or projectile metallic foreign bodies by Faghihi et al., industrial tool use by Yang et al., and digging during farm work and hammering on metal by Asencio et al.) [15, 18, 20]. In this study, the significant etiologies of OGI, either with or without accompanied endophthalmitis, were from activities producing high-velocity objects (lawn mowing, chiseling, and hammering). Therefore, while performing those at-risk activities, eye safety measures should be applied to successfully reduce the OGI and endophthalmitis incidence.

The relationship between the presence of IOFB and occurrence of endophthalmitis following OGI remains controversial [18–22]. Some studies confirmed an association between IOFB characteristics and the occurrence of endophthalmitis: higher risk in a wooden IOFB; and lower risk in a metallic foreign body with high velocity and high temperature as from explosion [23, 24]. However, Essex et al. indicated overall filthiness of OGI, rather than the presence of IOFB, was a risk of subsequent infection [17]. The inconclusive results may be attributed to differences in foreign body properties such as composition (metal or non-metal), contamination (clean or dirty), as well as the size of the causative material. In this study, a significant association between IOFB and endophthalmitis development was established as most IOFBs in this study were from lawn mowing-related gardening work, which was likely to be contaminated. Further evaluation in prospective studies may require elucidating a more evident relationship.

Apart from the IOFB, the time interval from injury to hospital and primary management were associated with the infection, consistent with other studies [18–20]. A large number of patients in this study presented to the hospital after 24 h following injury. The delayed presentation mostly related to an unawareness of the injury-related risks (until the development of infectious symptoms) by patients. This could be partly due to a high proportion of patients with self-sealed wounds which could appear nearly normal. Consequently, the delayed presentation allowed the amplification of microorganisms inside the eyeball. In this study, patients with self-sealing wounds had a greater number of IOFB, delayed presentation, and endophthalmitis. Therefore, patients with eye injuries, particularly those sustaining trauma from high-velocity objects, should receive prompt ophthalmic examinations with high concerns regarding the risk of intraocular infection, even when presenting with negligible symptoms.

Regarding wound location, some authors reported that OGI patients with corneal laceration had a higher risk of infection [18, 25]. Similarly, this study found that anterior wound location was a risk for occurrence of endophthalmitis. It is assumed that the more posterior wound location, particularly with intact conjunctiva, decreased the chance of the external pathogen inoculations.

To prevent endophthalmitis, an appropriate and timely antibiotic treatment is one of the essential strategies. Several methods of antibiotic administrations have been investigated in previous publications. Among these, effective managements in reducing the risk of post-traumatic infection include an immediate initiation of empirical prophylactic systemic antibiotics (either intravenous or oral administration) at initial presentation for all OGI and an injection of intravitreal antibiotics at the time of primary repair for high-risk patients [26–29]. According to our findings, signs of endophthalmitis development during treatments and complications following intravitreal antibiotics injections were not observed, thus supporting the aforementioned managements.

In post-traumatic endophthalmitis, a wide range of culture-positive rates (24% to 80%) have been published [10, 19, 20, 22, 30]. These variations may be attributable to differences in potential risks of infection between studies including setting and nature of injury, the use of prophylactic antibiotics, and the number of microorganisms and their susceptibility to antibiotics. A low positive culture rate (21%) in this study might be related to a low microbial load of infected agents. In addition, the prior treatment with antibiotics before presentation to this hospital and the absence of information from vitrectomy cassette fluid may partially contribute to a low detection rate.

Visual outcomes following post-traumatic endophthalmitis is another concerning issue [10, 20, 31, 32]. This study showed a similar trend of poorer visual prognosis of patients in the endophthalmitis group than the non-endophthalmitis group. In previous reports, initial VA, IOFB, retinal detachment, and microorganism virulence were the significant factors for visual outcomes in post-traumatic endophthalmitis patients. However, this study identified that the presence of RAPD and positive culture were prognostic factors for poor final VA, which may be due to their implications of the severity of ocular tissue destruction and the large number of infectious microorganisms responsible for endophthalmitis.

Limitations of this study were a retrospective design with insufficient information regarding injury settings in particular aspects. Inclusion of clinically diagnosed endophthalmitis patients, with negative cultures, may confound the results. However, all cases with intraocular infection in previous publications may not yield the positive cultures as well. Moreover, due to the detrimental consequences of endophthalmitis and the hazard of under-detection, the clinically diagnosed cases were more acceptable than the culture-proven cases in this study and other relevant studies [10, 17, 19]. The results add important information regarding risk factors and outcomes of post-traumatic endophthalmitis.

## Conclusion

This study shows that OGIs with corneal laceration, presence of IOFB, or delayed primary presentation were at high susceptibility for post-traumatic endophthalmitis. A poor visual prognosis for patients with OGI with accompanying endophthalmitis challenges the physician to provide timely and proper treatment. Therefore, an immediate institution of prophylactic systemic antibiotics for suspicious OGI patients and intravitreal antibiotics for high-risk groups is recommended.

## Data Availability

The datasets used and/or analyzed during the current study are available from the corresponding author on reasonable request.

## Abbreviations

BETT: The Birmingham Eye Trauma Terminology System
CI: Confidence interval
IOFB: Intraocular foreign body
IQR: Interquartile range
logMAR: Logarithm of the minimum angle of resolution
NPL: No perception of light
OR: Odds ratio
OGI: Open globe injury
PPV: Pars plana vitrectomy
RAPD: Relative afferent pupillary defect
SD: Standard deviation
VA: Visual acuity
